# Wolff-Parkinson-White Syndrome Mimics a Conduction Disease

**DOI:** 10.1155/2014/789537

**Published:** 2014-07-09

**Authors:** S. Marrakchi, I. Kammoun, S. Kachboura

**Affiliations:** Department of Cardiology, Abderrahmen Mami Hospital, Medical School of Tunis, Faculty of Medicine of Tunis Ariana, Tunisia

## Abstract

*Background*. It is important to recognise Wolff-Parkinson-White (WPW) syndrome in electrocardiograms (ECG), as it may mimic ischaemic heart disease, ventricular hypertrophy, and bundle branch block. Recognising WPW syndrome allows for risk stratification, the identification of associated conditions, and the institution of appropriate management. *Objective*. The present case showed that electrophysiological study is indicated in patients with abnormal ECG and syncope. *Case Report*. A 40-year-old man with Wolff-Parkinson-White syndrome was presented to emergency with syncope. A baseline ECG was a complete right branch block and posterior left hemiblock. He was admitted to the cardiac care unit for pacemaker implantation. The atypical figure of complete right branch block and posterior left hemiblock was thought to be a “false positive” of conduction abnormality. But the long anterograde refractory period of the both accessory pathway and atrioventricular conduction may cause difficulty in diagnosing Wolff-Parkinson-White syndrome, *Conclusion*. A Wolff-Parkinson-White Syndrome may mimic a conduction disease. No reliable algorithm exists for making an ECG diagnosis of a preexcitation syndrome with conduction disorders. This can lead to diagnostic and therapeutic dilemmas in the context of syncope.

## 1. Introduction

Wolff-Parkinson-White (WPW) syndrome is a conduction disturbance in which atrial impulses are transmitted to the ventricle by an accessory pathway besides normal atrioventricular conduction. The result of these multiple fronts of depolarization is a short PR interval, the delta wave, and a widened QRS complex. The preexcitation syndrome (PS) is diagnosed by the surface ECG in sinus rhythm with a typical pattern associating a short PR interval (<0.12 s in adults) and a widening of QRS complex with a delta wave [1, 2]. Patients with WPW syndrome may usually present palpitation, syncope, and sudden death. Pattern of PS is noted among 0.1 to 0.5% of the population [3]. The pattern is dependent not only on the location of accessory pathway (AP), but also on the properties of atrioventricular (AV) node and His-Purkinje system, and there is a wide spectrum of ECG types encountered. Spontaneous normalization of ECG with intermittent preexcitation is reported in 20% to 30% of WPW; dynamic QRS variations in WPW syndrome were noted several years ago [4].

This case reported unexplicated syncope with unusual patterns of PS related to an atrioventricular accessory pathway (AP) was identified at electrophysiological study.

## 2. Case Report

**Figure 1:**
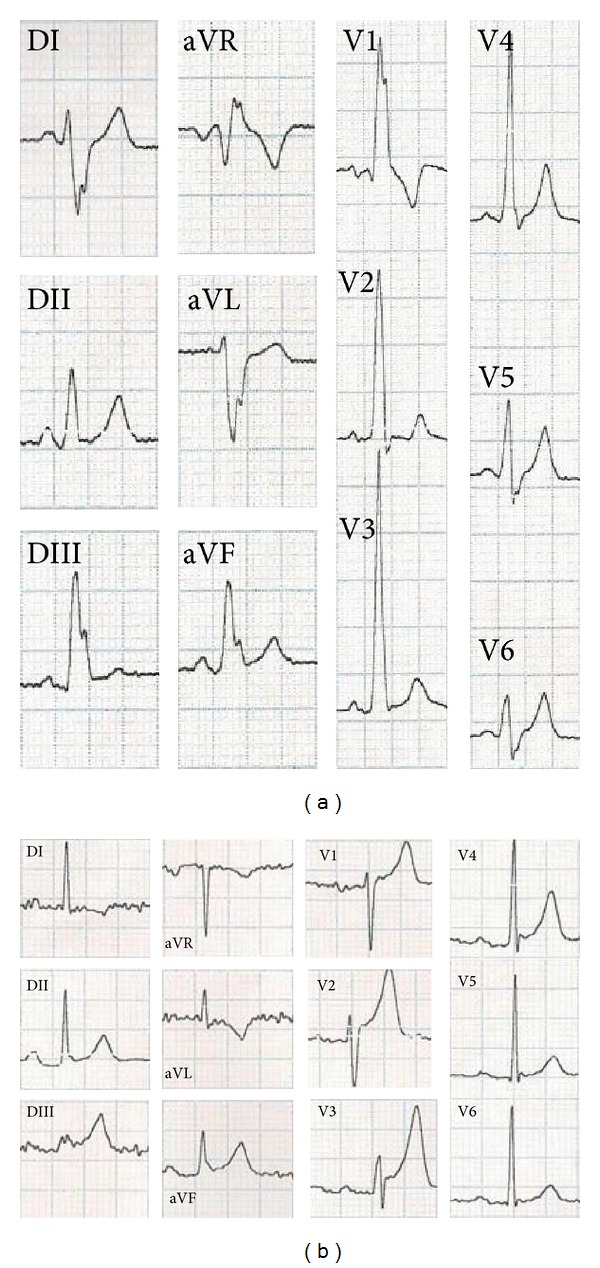
(a) Regular sinus rhythm at 70 beats per minute, a wide QRS and PR interval was 120 ms (b) post ablation we show a normal QRS.

This case is about a 40-year-old man who was presented to emergency with syncope that happened at work. He had no prior history of cardiac disease. The physical examination was normal. The ECG showed a regular sinus rhythm at 70 beats per minute, a complete right branch block, and posterior left hemiblock (Figure 1), and PR interval was 120 ms. Transthoracic echocardiography revealed normal echocardiographic findings with normal left ventricular systolic function.

**Figure 2:**
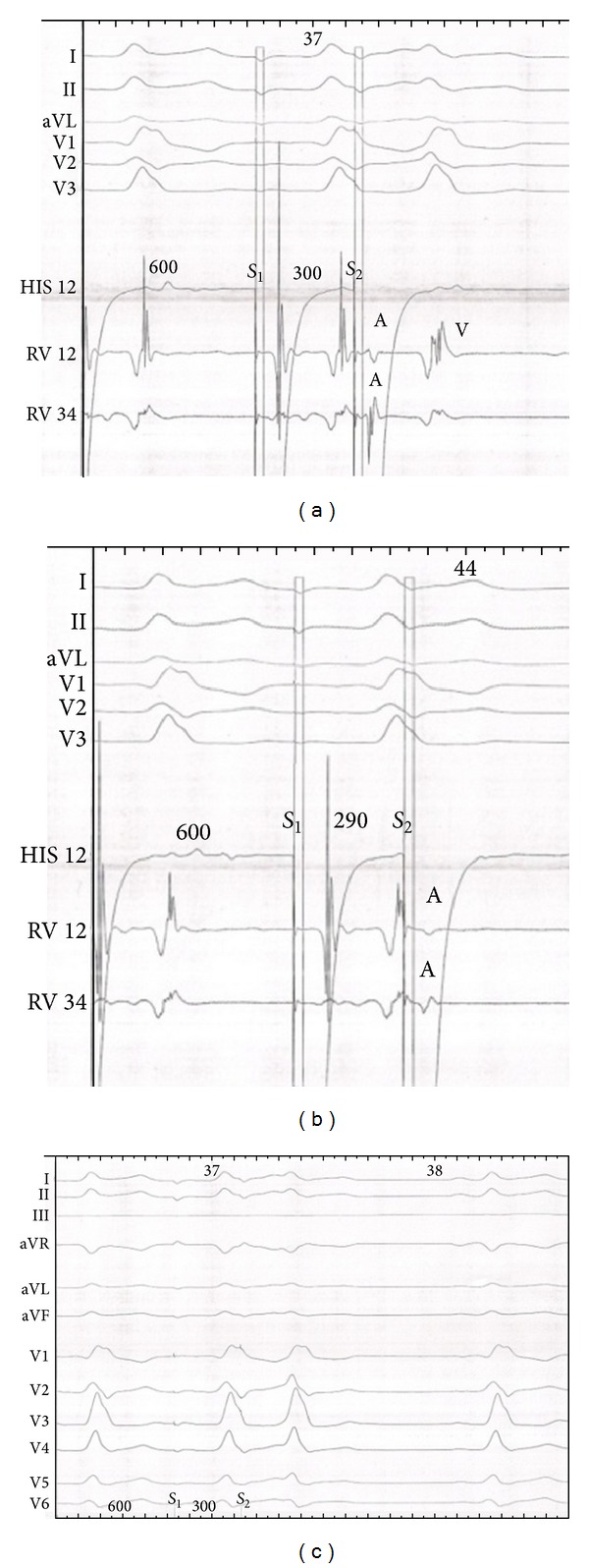
In atrial pacing, effective accessory pathway refractory period was 290 ms effective ((a) and (b)). The QRS had the same morphology during atrial pacing (c).

**Figure 3:**
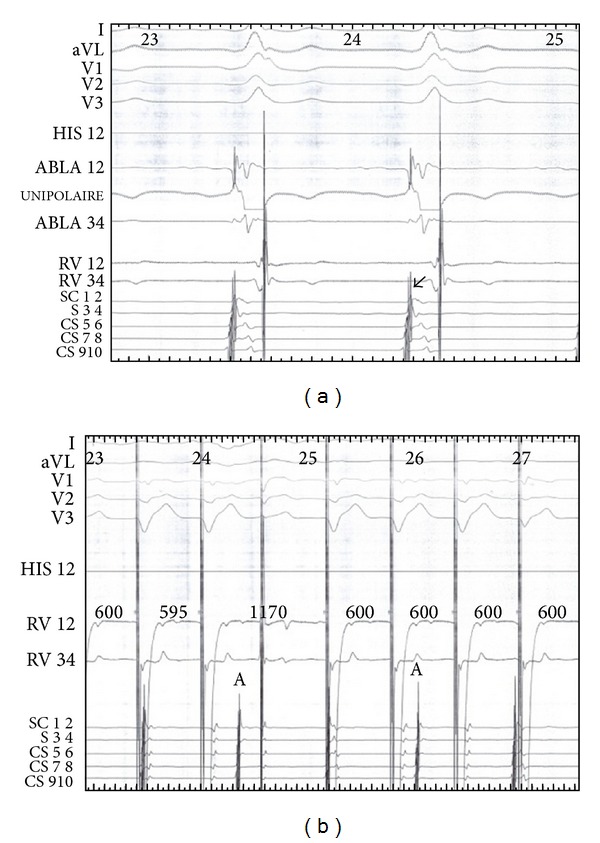
The electrophysiology study showed a shorter AV at the distal electrode of decapolar (a) with dissociation atrioventricular conduction in retrograde (b). This figure confirmed the diagnosis of a left accessory bypass with only anterograde conduction.

**Figure 4:**
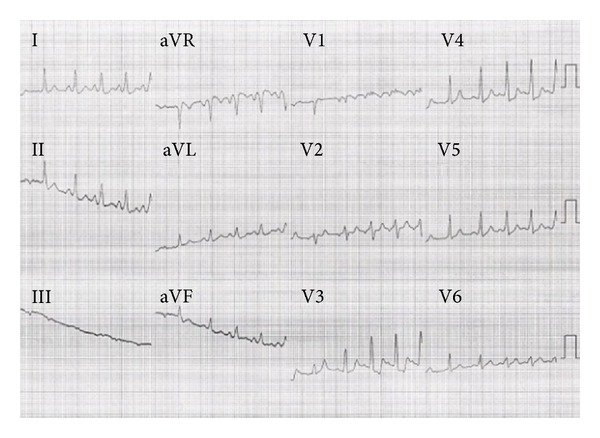
ECG after using isuprel: the use of isuprel showed the classic figure of Wolff-Parkinson-White syndrome with a typical QRS with slurred initial QRS.

**Figure 5:**
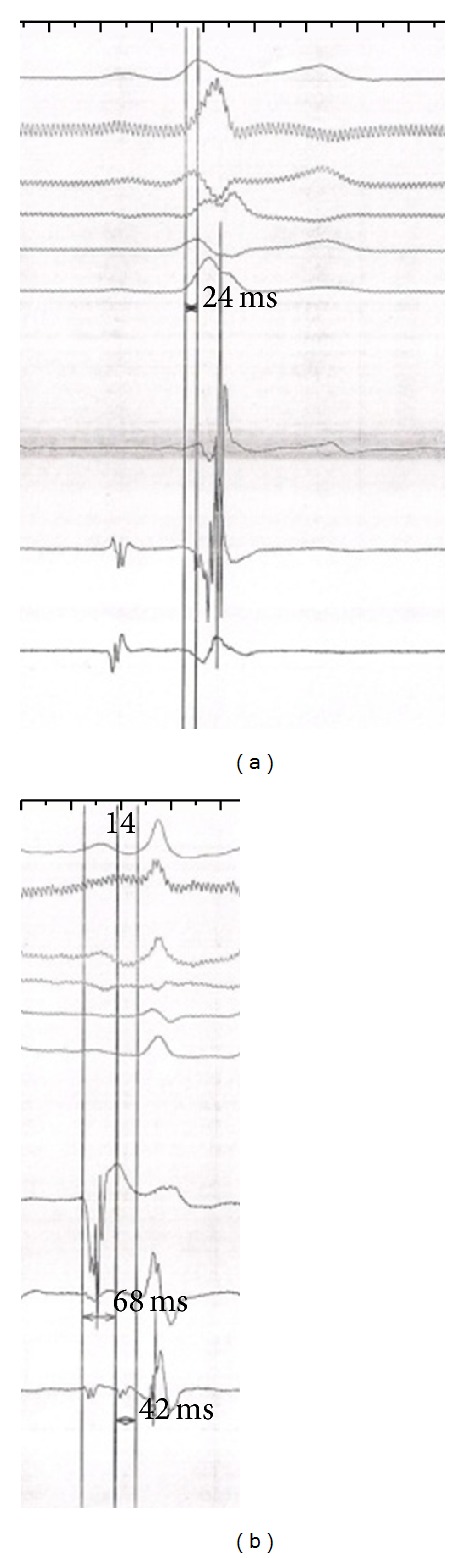
After the use of isuprel ((b) HV was 42 ms), HV interval was longer than the basal state ((a) HV was −24 ms). The isuprel accelerated atrioventricular conduction.

**Figure 6:**
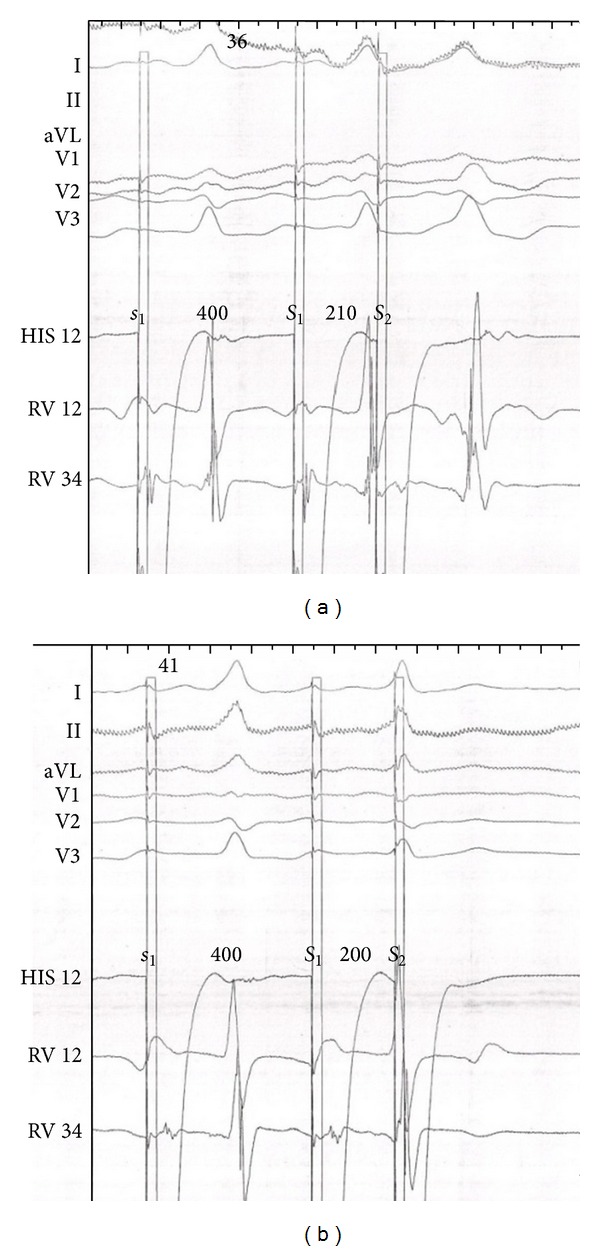
In atrial pacing, effective accessory pathway refractory period was 290 ms effective ((a) and (b)). The QRS had the same morphology during atrial pacing.

**Figure 7:**
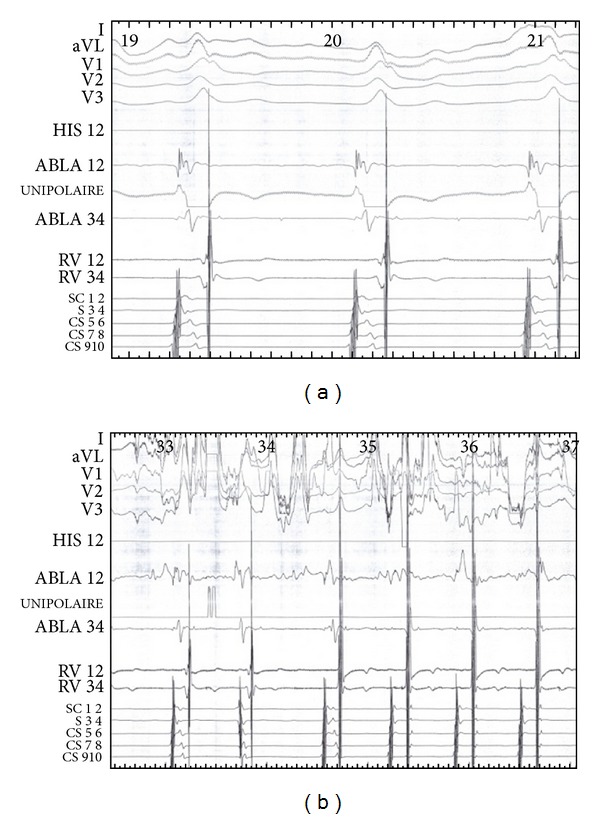
(a) Electrophysiology signal before the ablation. (b) The distal coronary sinus signal showed a separated ventricular signal from atria signal during ablation. The successful left accessory bypass ablation persisted.

Electrophysiological study (EPS) was performed by intracardiac route in this patient with unexplained syncope and atypical ECG. We recorded a regular sinus rhythm, an AH interval was 150 ms, and HV interval was −24 ms. Programmed atrial stimulation was performed in the basal state and showed an effective atrial refractory period of right atrium at 200 ms and effective refractory period of accessory tract was 300 ms (Figure 2). The QRS had the same morphology during atrial pacing. We found a dissociation atrioventricular conduction in retrograde. We inserted a catheter of exploration in coronary sinus; we recorded a shorter AV at the distal electrode of decapolar that confirmed the diagnosis of a left accessory bypass (Figure 3). The use of isoproterenol (isuprel) showed a classic Wolff-Parkinson-White syndrome with slurred initial QRS. This ECG showed fusion of antergrade conduction from both the left sided accessory pathway (AP) and AV node (Figure 4). AH interval with isuprel was 100 ms and HV interval was 42 ms. This was explicated by an accelerated atrioventricular conduction with isuprel in addition to accessory pathway (Figure 5). The effective refractory period of accessory pathway was 210 ms with isuprel (Figure 6). We supposed that atrial fibrillation caused ventricular fibrillation responsible for syncope. Thus, we had decided to ablate the accessory pathway (Figure 7).

After accessory pathway ablation, the patient was in nodal AV block with QRS complexes of normal duration (0.10 s). In Atrial pacing, an anterograde Wenckebach cycle length was 130/BAT (suprahisian AV block). The patient became asymptomatic.

## 3. Discussion

Unapparent preexcitation syndrome was defined as a normal ECG at the time at electrophysiology which can be related to a masked preexcitation or an intermittent preexcitation. Masked preexcitation was defined as overt antegrade conduction over AP masked by the normal AV conduction. These patients have a minimal preexcitation on the ECG [5].

We reported a case of a patient with WPW and syncope. The preablation ECG showed fusion of antegrade conduction from both the left sided accessory pathway (AP) and AV node with predominance from left sided accessory pathway. Therefore, the ECG showed atypical presentation of preexcitation that is considered as rare in PS. This patient was studied because he complained of syncope. Similar findings could be expected in apparently asymptomatic subjects; systematic ECG in subjects at risk of arrhythmias as athletes is probably not sufficient to eliminate the presence of a masked electrical abnormality as a PS sometimes at risk of serious events. The most frequent unusual ECG of PS was the masked pattern of PS, associated with minor signs of preexcitation retrospectively visible when ECG became slightly different after AP ablation [5]. Although the ECG mimics RBBB, a more careful inspection of the baseline ECG indeed reveals a small delta wave and relatively short PR interval. A differential diagnosis of AP could be made after careful analysis [6].

It is important to recognize Wolff-Parkinson-White (WPW) syndrome in electrocardiograms (ECG), as it may mimic ischaemic heart disease, ventricular hypertrophy, and bundle branch block. In addition, ECG can aid in the localization of the accessory pathway. Recognizing WPW syndrome allows for risk stratification [1, 7], the identification of associated conditions, and the institution of appropriate management. More than 50% of APs are located at the left free wall, 5%–10% at the anteroseptum, 20%–30% at the posteroseptum, and 10%–20% at the right free wall [8]. In patients with an antegradely conducting AP, ventricular activation during sinus rhythm occurs simultaneously via both the AP and the atrioventricular (AV) node, resulting in a fusion complex. APs may be classified into several types [9]. Manifest APs are those that conduct more rapidly in the antegrade direction than the AV node, resulting in a discernible delta wave on the surface ECG. Concealed APs conduct only in the retrograde direction, and no delta wave is documented in the ECG. Several algorithms have been developed to localize the site of APs from the surface ECG [10, 11]. In our case, the anterograde conduction in accessory tract and atrioventricular node both slowly explain this ECG figure. Charron et al. [12] reported a particular electrocardiographic pattern in four members of the same family with conduction defects (RBBB and occasionally left anterior hemiblock), short PR interval, pseudoappearance of atrial hypertrophy, and occasionally sinus dysfunction or supraventricular extrasystole. This clinical entity is related to R302Q mutation of the *γ*2 subunit producing AMP protein kinase, coded by the gene PRKAG2, but, in our case, there was not a similar case in his family.

In conclusion, a Wolff-Parkinson-White syndrome may mimic a conduction disease and this can lead to diagnostic and therapeutic dilemmas in the context of syncope.
